# A Prospective Observational Study to Assess Attachment Representations With Regard to Neurocognitive and Behavioral Outcomes in Children Born Very Prematurely in the Loire Infant Follow-Up Team (LIFT Cohort)

**DOI:** 10.3389/fped.2022.896103

**Published:** 2022-07-13

**Authors:** Elise Riquin, Ramona Sandnes, Fabien Bacro, Aubeline Vinay, Raphaële Miljkovitch, Valérie Rouger, Josué Rakotonjanahary, Géraldine Gascoin, Jean-Baptiste Müller

**Affiliations:** ^1^Department of Child and Adolescent Psychiatry, University Hospital of Angers, Angers, France; ^2^CHU Angers, University of Angers, Angers, France; ^3^Fondation de Santé des Étudiants de France, Clinique de Sablé sur Sarthe, Sablé sur Sarthe, France; ^4^Faculty of Psychology, Centre de Recherche en Education de Nantes (CREN – EA 2661), University of Nantes, Nantes, France; ^5^Départements d’Enseignement LLSH - DEP ENS LLSH Psychologie, UFR Lettres Langues et Sciences Humaines – LLSH, Angers, France; ^6^Laboratoire Paragraphe, Université Paris 8, Saint Denis, France; ^7^Loire Infant Follow-Up Team (LIFT) Network, Pays de Loire, France; ^8^Department of Pediatric Oncology, University Hospital, Angers, France; ^9^Department of Neonatal Medicine, Toulouse University Hospital, Toulouse, France; ^10^Department of Neonatal Medicine, Nantes University Hospital, Nantes, France; ^11^National Institute of Health and Medical Research CIC004, Nantes University Hospital, Nantes, France

**Keywords:** prematurity, development, attachment, child, infant

## Abstract

**Context and purpose:**

Prematurity is a situation that can disrupt parent-child interactions. We hypothesize that establishing relationships with parents in a context of extreme prematurity can alter the development of secure attachment representations in the child. Furthermore, we hypothesize that secure maternal representations and their possible interactions with prematurity factors prevent the development of insecure or disorganized attachment in the child. In addition, maternal representations and their possible interactions with factors related to prematurity may prevent or accentuate the development of an insecure or disorganized attachment in the child.

**Methods and analysis:**

This is a longitudinal, prospective, exploratory, and bi-centric study. Children born in the neonatal intensive care units of Angers or Nantes University Hospitals with a gestational age of up to 28 weeks will be included in the study. The main objective is to describe the attachment representations at 3 and 5 years through the Attachment Story Completion Task scales and to analyze them in regard to the children’s neurocognitive and behavioral outcomes as well as maternal attachment and mental health.

**Ethics:**

The study file received a favorable opinion for the implementation of this research on February 18, 2020 - ID-RCB no. 2019-A03352-55 (File 2-20-007 id6699) 2°HPS. This study has received authorization from the French Data Protection Authority (CNIL) under no. 920229.

**Discussion:**

A better understanding of attachment representations in extreme prematurity and their possible associations with children’s neurocognitive and behavioral outcomes as well as maternal attachment and mental health could pave the way for individualized care at an early stage, or even interventions during the neonatal period to improve the outcome of these vulnerable newborns.

**Trial registration:**

[ClinicalTrials.gov], identifier [NCT04304846].

## Introduction

The long-term consequences of prematurity include behavioral and cognitive impairments that may manifest at school age as learning disabilities (1, 2). Indeed, preterm births are also associated with a high risk of neurodevelopmental disabilities (3, 4).

Term or preterm newborns aim to come into contact with those around them and build close relationships with their caregivers (5). These attachment relationships are established gradually and, from the end of the first year, children exhibit individual attachment behavior patterns, depending on the quality of interactions and responses given to them by adults (6).

Prematurity can disrupt parent-child interactions (7, 8). The physiological characteristics of preterm infants attenuate and modify their signals of stress. Visual interactions are shorter, they struggle to stay focused, and their reactions are less visible than those of full-term infants (9). In this stressful and critical period, maternal sensitivity and availability may also be impaired (10). Parental stress, as well as post-traumatic stress disorders, can disrupt the emotional sense of parenthood (11, 12). Thus, in these situations, changes in the quality of mother-child interactions have been reported, with more controlling maternal behavior (13) and the development of behavioral impairments in children (13). It has also been shown that inadequate maternal caregiving is associated with relational withdrawal of the child, especially in the case of prematurity (14).

What is more, maternal attachment representations have been associated not only with children’s developmental outcomes (15–17) but also with children’s brain development (18), and there may be an intergenerational transmission of attachment (19).

In the context of prematurity, literature on the intergenerational transmission of attachment is scarce. To our knowledge, no study has explored this attachment pathway in the context of extreme prematurity with regards to maternal mental health and child neuro-development.

Firstly, we hypothesize that the experience of establishing a relationship with parents in a context of extreme prematurity can alter the development of secure attachment representations in the child. Secondly, we also hypothesize that the potential disorganization of children’s attachment representations is associated with clinical and environmental factors, as well as with maternal attachment representations, the emotional state of parents, and neuro-developmental complications (motor, cognitive, and behavioral) for the child.

## Methods and Analysis

Aims:

The primary aim of this study is the description of attachment representations at 3 and 5 years of age, and their links with neurocognitive and behavioral outcomes in children born very prematurely.

The secondary aims are to test:

- The link between attachment representations at 3 and 5 years of age and perinatal and/or prenatal and socio-environmental factors in children born very prematurely.
- The link between maternal attachment representations and:
  ० children’s attachment representations at 3 and 5 years in children born very prematurely; and
  ० neurocognitive assessment at 3 and 5 years in children born very prematurely.

Design of the study and characteristics of the participants:

This is a longitudinal, prospective, exploratory, and bi-centric study. The inclusion criteria are: singleton infants born at less than 29 weeks of gestation in the neonatal intensive care unit (NICU) of Angers and Nantes University Hospitals included in the regional monitoring network for vulnerable newborns (Loire Infant Follow-up Team, LIFT Cohort) and with a clinical evaluation planned at 3 years of age in Angers or Nantes Hospital, with an informed consent dated and signed by the parents. The exclusion criteria are: being a child with severe neurocognitive impairment or a severe autism disorder in the 2-year LIFT follow-up evaluation.

Neonatal characteristics of the cohort are presented in Table 1.

**TABLE 1:** Selected baseline characteristics of the preterm children included in the cohort.

Variable	*n* (%) or mean ± sd [min; max]
Age (years) mean, sd [min; max]	31.3 ± 5.6 [20;42]
Obesity (BMI > 30), n (%)	16 (19.8)
Number of children at home, mean, sd [min; max]	1.7 ± 1.2 [1;5]
Low parents’ socioeconomic level, n (%)	17 (23.6)
Intermediate parents’ socioeconomic level, n (%)	38 (52.7)
High parents’ socioeconomic level, n (%)	17 (23.6)
Assisted Conception Medically Assisted Reproduction, n (%)	10 (12.3)
Isolated IUGR, n (%)	20 (24.7)
IUGR associated to maternal hypertensive pathology, n (%)	12 (14.8)
Threatening Premature delivery, n (%)	46 (56.8)
Antenatal corticotherapy, n (%)	73 (90.1)
- 1 dose	27 (37.0)
- 2 doses	45 (61.6)
Hypertension during pregnancy, n (%)	22 (27.2)
Magnesium sulfate, n (%)	50 (61.7)
C-section, n (%)	45 (55.6)
Male Gender, n (%)	41 (50.6)
Gestational age, mean ± sd [min;max]	26.5 ± 1.9 [24.0;28.6]
24-25	31 (38.2)
26-27	31 (38.2)
28	19 (23.5)
Birth weight (g) mean ± sd [min;max]	865.1 ± 272.7 [450;1420]
- *Z*-score of birth weight	−0.3 ± 1.1 [−3.3;1.9]
- *Z*-score < -2sd	23 (28.4)
HC (cm) mean ± sd [min;max]	24.2 ± 2.2 [20.2;28.0]
- *Z*-score HC	−0.5 ± 0.9 [−2.8;1.8]
- *Z*-score HC < -2sd	22 (27.2)
Ligature of ductus arteriosus, n (%)	2 (2.4)
Ulcerative necrotizing enterocolitis, n (%)	2 (2.4)
Intraventricular hemorrhage grade 3/4 or Periventricular Leukomalacia, n (%)	7 (8.6)
Bronchopulmonary dysplasia (support at 36 GW), n (%)	45 (55.0)
Outborn delivery, n (%)	8 (10.0)
Intubation at birth, n (%)	43 (53.1)
Apgar score at 5 min <7, n (%)	27 (33.3)

*BMI, Body Mass Index; n, number; %, percentage; g, grams; HC, head circumference; min, minimum; max, maximum; O2, oxygen therapy; SD, Standard Deviation; GW, Gestational Week.*

Description of materials:

Data collected through the usual LIFT follow-up assessment:

- Parental and socioeconomic data:

Parental data comprised maternal age, number of children at home, parents’ socioeconomic level. The socioeconomic data consisted of the socioeconomic level and eligibility for social security benefits of those with low incomes. The socioeconomic level took into account the parent with the most highly-rated job according to a scale based on the official classification developed by the INSEE institute.

- Obstetrical data:

Obstetrical data comprised the context of Medically Assisted Reproduction, Intra-Uterine Growth Retardation (IUGR), threatening premature delivery, use of assisted conception, antenatal corticotherapy, C-section, the existence of maternal hypertensive disorders during pregnancy, and the use of antenatal corticotherapy or magnesium sulfate.

- Perinatal data:

Perinatal data comprised date of birth, gender, gestational age (GA), birth weight, and head circumference (HC). Birth weight and HC were expressed in *Z*-scores according to Olsen standards. Data on neonatal morbidity, such as criteria used in the context of the LIFT follow-up, including bronchopulmonary dysplasia at 36 WG, grade 3-4 intraventricular hemorrhage, periventricular leukomalacia, grade 2-3 necrotizing enterocolitis, and persistence of the arterial duct treated, were also collected.

Follow-up data: Questionnaires included in the LIFT cohort follow-up and specific data that will be collected for this study will be analyzed and are described in Table 2. TheAges and Stages Questionnaire (ASQ) (20, 21), the Strengths and Difficulties Questionnaire (SDQ) (22, 23), the Global School Adaptation (GSA) (24, 25), the Attachment Story Completion Task (ASCT) (26, 27), the Attachment Multiple Model Interview (AMMI) (28), the Parenting Stress Index (PSI) (29–31), and the Perinatal Posttraumatic Stress Disorder (PTSD) Questionnaire (PPQ) (29, 32) were used in the study.

**FIGURE 1:**
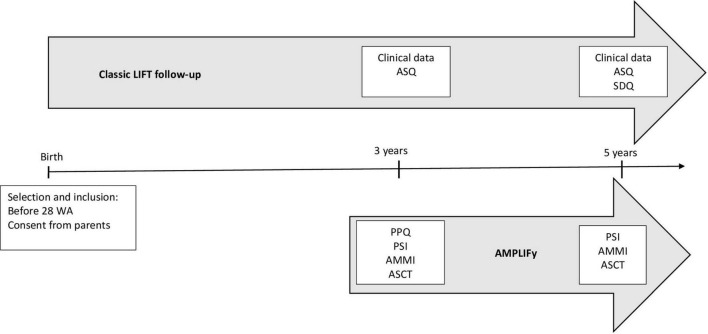
Study time schedule. AMMI: Attachment Multiple Model Interview; PSI: Parental Stress Index; PPQ: Perinatal Post-Traumatic Stress Disorder Questionnaire; LIFT: Longitudinal study of preterm infants in the Pays de la Loire region of France; ASQ: Ages and Stages Questionnaires; SDQ: Strength and Difficulties Questionnaire; GSA: Global School Adaptation; WA: Weeks of Amenorrhea; AMPLIFy: AttachMent Preterm LIFy.

The outcome measures are described in Table 3.
Course of the study:
The course of the study is presented in Figure 1.

**TABLE 2:** Description of assessment tools.

Classic LIFT measures	
**Clinical categorization carried out by the LIFT referring doctor**	
Parental questionnaire Ages and Stages Questionnaire (ASQ)	Neurodevelopmental outcome is assessed with the parent-completed “Ages and Stages Questionnaire” (ASQ) (20). The ASQ assesses development in the following five areas: communication, gross motor, fine motor, problem solving and personal-social skills. The maximum overall ASQ score is 300 and a score below 185 is considered non-optimal (21).
Parental questionnaire Strengths and Difficulties Questionnaire (SDQ)	The SDQ (22) is a screening questionnaire for measuring parents’ perceptions of their child’s prosocial and problem behaviors. It includes 25 items on a three-level scale ranging from 0 (not true) to 2 (very true). The items are split into five sub-scales that each comprise five items: emotional problems, behavioral problems, hyperactivity/inattention, peer relations, and prosocial behavior. A total difficulties score (between 0 and 40) can be calculated by adding up scores on all the scales except the prosocial scale. In a normal french population, a cutoff at 17 represents the 90th percentile (23).
Teacher’s questionnaire (GSA)	The GSA score was originally defined as a tool to be used by teachers to assess children’s abilities and behavior in the classroom (24). At 5 years of age, the questionnaire is given to parents of children monitored through the LIFT network, who then forward it to the teachers. Six questions investigate linguistic competence (school conversation, participation, pertinence, vocabulary, syntax and pronunciation, and understanding), and five questions investigate non-verbal abilities (memory, arithmetic, capacity for logic, manual ability, and gross motor coordination). Eight questions relate to behavior in the classroom (respect for classroom rules, attention, independence when faced with a task, speed of task execution, work organization, self-confidence, the ability to keep up with the pace of the classroom, and tiredness). The final question asks the teacher to provide their prognosis for the child’s future adaptation to school life. The answer to each question is assigned a score between 1 and 3, with higher values representing better abilities. The total score is calculated by adding up the points from the 20 questions (range of potential scores: 20-60). A higher score corresponds to better adaptation by the child to school life. The threshold value for a positive evaluation of a child’s adaptation to school life was previously defined as a score greater than 45 (24, 25).
**AMPLIFy measures**	
Attachment Story Completion Task (ASCT)	The ASCT (26) consists of a series of story stems with narratives that are designed to elicit children’s attachment representations and feelings. The task is both narrated and enacted with small human figures each one of which is initially introduced as a member of a family (mother, father, daughter, son, grandmother, etc.). In addition, there are simple props related to each story stem. The task is suitable for preschoolers as young as 3 years of age because narration can be supplemented by action. A coding procedure was developed in French by Miljkovitch et al. to assess the level of security, deactivation (avoidance), hyperactivation (resistance), and disorganization of the attachment system (27). The child is given a score on each of these scales by correlating their item scores with 4 attachment prototypes (secure, deactivating, hyperactivating, and disorganized).
Attachment Multiple Model Interview (AMMI)	The AMMI is a semi-structured interview designed to assess attachment representations of relationships with different attachment figures, such as their mother, father and romantic partners (28). The coding system enables the level of security, inhibition, hyperactivation and disorganization to be assessed for each of the relationships explored. The participant is given a score on each of these scales for each of the relationships assessed. Scores range from 0 to 8, except for the disorganization scale, which ranges from 0 to 16. The AMMI is associated with the individual’s past attachments with his/her attachment figures (28).
Parental Stress Index (PSI)	The PSI (29, 30) is a self-administered questionnaire assessing difficulties faced by parents of children aged 0 to 10 years in their parental role. The PSI measures perceived parenting stress. It is a 5-point Likert-type scale including 120 items on two domains: the child and parent characteristics domain (101 items, 47 and 54 items in the child and parent domain, respectively). Its validation and reliability have been substantiated. It is recommended as a screening and interpretive tool for evaluating the state of the parenting system as it is perceived by the parent. Its aim is to offer interpretive guidelines to help identify issues that may lead to problems in the child’s or parent’s behavior that may aggravate parenting. As such, PSI scores are not to be used as a diagnostic tool, but rather as working hypotheses that health professionals may use to assist individual parents. The instrument consists of a Child Domain with 6 subscales (Distractibility/Hyperactivity, Adaptability, Reinforces Parent, Demandingness, Mood, and Acceptability) and a Parent Domain with 7 subscales (Competence, Isolation, Attachment, Health, Role Restriction, Depression, and Spouse/Parenting partner relationship). Each of these stressors represents a sub-scale on which the higher the value, the higher the stress. The different sub-scales provide good internal consistency, as well as good temporal stability (30, 31).
Perinatal Post-Traumatic Stress Disorder (PTSD) Questionnaire (PPQ)	The PPQ (32) is a 14-item self-administered questionnaire specially designed for parents of high-risk perinatal children. Mothers answer either yes or no to each of the 14 items that form the three elements of PTSD: intrusive memories, avoidance behaviors, and symptoms of hyperarousal. The score range is from 0 to 42. A score higher than 6 may be considered as high risk for a PTSD diagnosis according to the DSM-IV (17,19).

*AMMI, Attachment Multiple Model Interview; PSI, Parental Stress Index; PPQ, Perinatal Post-Traumatic Stress Disorder Questionnaire; LIFT, Longitudinal study of preterm infants in the Pays de la Loire region of France; ASQ, Ages and Stages Questionnaires; SDQ, Strength and Difficulties Questionnaire; GSA, Global School Adaptation.*

**TABLE 3:** Outcome measures.

Objectives	Outcomes measures and timepoint(s) of evaluation
**Primary aim**	
Description of attachment representations compared to neurocognitive and behavioral outcomes in children born very prematurely. [Time Frame: Three and five years after preterm birth]	Attachment Story Completion Task
**Secondary aims**	
Description of attachment representations compared to perinatal and/or prenatal and socio-environmental factors in children born very prematurely. [Time Frame: Three and five years after preterm birth]	Child’s medical history to be completed (3 to 5 years of age) AMMI (3 and 5 years) PSI (3 and 5 years) PPQ (3 years)
Description of attachment representations in children born very prematurely compared to maternal attachment representations. [Time Frame: Three and five years after preterm birth]	+ classic LIFT cohort follow-up: Clinical categorization carried out by the LIFT cohort referring doctor (3 and 5 years old)
Neuro-cognitive and behavioral assessment in children born very prematurely compared to maternal attachment representations. [Time Frame: Three and five years after preterm birth]	Parental questionnaire ASQ (3 and 5 years) Parental questionnaire SDQ (5 years) Teacher’s questionnaire GSA (5 years)

*AMMI, Attachment Multiple Model Interview; PSI, Parental Stress Index; PPQ, Perinatal Post-Traumatic Stress Disorder Questionnaire; ASQ, Ages and Stages Questionnaires; SDQ, Strength and Difficulties Questionnaire; GSA, Global School Adaptation; LIFT, Longitudinal study of preterm infants in the Pays de la Loire region of France.*

Eligible patients will be identified in the LIFT cohort. The information letter will be sent to parents by post before the follow-up visit at 3 years old. A study investigator then completes the inclusion process by phone (collects additional information, answers questions from the parents, and obtains their express consent). The questionnaires (PSI and PPQ) will be sent by post one month before the appointment scheduled for the visit at 3 years of age.

Visit at 3 years of age:

The standard LIFT cohort follow-up will be performed. As part of the follow-up, prior to the appointment parents will receive the ASQ questionnaire that they will complete before the appointment and return to the LIFT cohort administration department in an enclosed prepaid envelope (one month before), specifying the date on which the questionnaire was completed.

Patients will be asked to provide consent relating to image rights before the interviews are carried out. Data from the follow-up at 3 years, as planned under the LIFT cohort, will be extracted and included in the AMPLIFy study case report forms.

Additional investigations will be carried out as part of this study: collection of the completed PSI and PPQ questionnaires, the AMMI, a 30-min interview (recorded) to assess the mother’s attachment representations to her own mother, father, and spouse and the ASCT, a story-completion task with the child (filmed) in the presence of a second accompanying adult, during which the child is asked to complete five story beginnings.

Visit at 5 years:

The standard LIFT cohort follow-up will be performed. As part of this follow-up, before the parent’s appointment the ASQ, the SDQ, and GSA will be filled out by parents. Questionnaire results will be available for the consultation at 5 years.

Data from the follow-up at 5 years, planned as part of the LIFT cohort, will be extracted and included in the AMPLIFy study case report forms.

Additional investigations will be carried out as part of this study: collection of the PSI and PPQ questionnaires (if not completed during the visit at 3 years), the AMMI, a 30-min interview (recorded) with the child’s mother to assess the mother’s attachment representations to her own mother, father, and spouse and the child’s attachment representations with the ASCT.

The inclusion and participation periods will each last 2 years and the study (inclusion + participation periods) will last 4 years in total.

**FIGURE 2:**
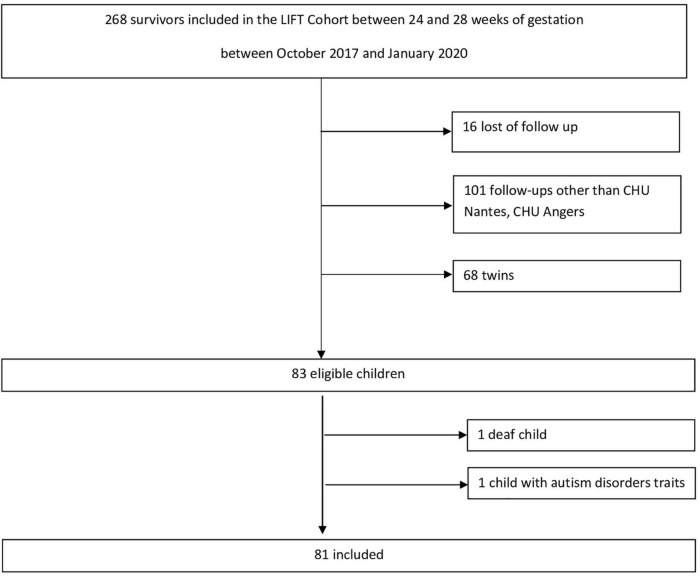
Study flow chart.

Sample size calculation:

As this is a pilot study exploring the attachment representations in children born very prematurely, the size of the sample to be analyzed was determined using the data in the LIFT cohort database. Data from centers participating in the study were used to determine the number of participants who were eligible for inclusion at these centers and to estimate the total number of participants eligible for the entire study. Figure 2 shows the details regarding the number of eligible patients.

A total of 83 children are considered as eligible for inclusion in the two centers and will be screened for study recruitment. Participation will be offered to the parents (or the legal guardians). Assuming the occurrence of a non-participation rate in the study (due to refusal or for another reason) and an incomplete data rate for an estimated total of 25% of the eligible sample, the number of children with complete analyzable data is estimated at 63 in total. This aligns with the desired sample sizes recommended for pilot studies reported in the literature (33, 34). Participation will be offered to the parents (or the legal guardians) of 83 children considered eligible for inclusion. Consistent with the exploratory nature of this pilot study, we did not perform a statistical power analysis. Power calculations are beyond the scope of a pilot study (35). We will use a confidence interval approach to interpret the results (36). After taking into account exclusion criteria, a total number of 81 children will be included in the study.

Statistical analysis design: A flow chart will be used to depict the screening and inclusion of children included in the analysis. A descriptive analysis of the characteristics of the children included in the study will be carried out.

Descriptive analyses will be presented for all of the data collected about the children included in the study, as well as the data relating to the parents. The qualitative variables will be studied in terms of frequency and percentage. Quantitative variables will be analyzed in terms of absolute scores, medians, interquartile ranges (or extreme values), averages, standard deviations, and/or 95% average confidence intervals.

For each Q-sort score derived from the ASCT (secure, deactivating, hyperactivating, and disorganized), the average of each score recorded at 3 years and again at 5 years will be used to describe children’s attachment representations within the population of children born extremely prematurely (27).

The analysis will take place on an exploratory basis by studying the factors associated with the children’s attachment representations at 3 and 5 years old. The factors associated with each Q-score will be studied using a univariate linear regression model. A separate model will be performed for each Q-score. Multivariate analyses will be also carried out on an exploratory basis (one multivariate model for each Q-score). The following different factors will be considered (37), including specifically: neurocognitive development (using the score from the GSA questionnaire as an absolute value and the score from the ASQ questionnaire expressed as a score <285 or not); behavioral development (SDQ score between 17 and 40 or not); perinatal, prenatal, and socioenvironmental factors (term of pregnancy, birth weight and length as a z-score, sex, age of the mother, gravidity, parity); comorbidity at birth (composite criterion used in the context of the LIFT follow-up: bronchopulmonary dysplasia at 36 WG, grade 3-4 intraventricular hemorrhage, periventricular leukomalacia, grade 2-3 necrotizing enterocolitis, ligature of ductus arteriosus); the socioeconomic status and level of education of the parents; the use of medically assisted reproduction; the parents’ marital status (separated or not). The following factors will also be taken into account: parental posttraumatic stress, using the score from the PPQ questionnaire (score greater than or equal to 6), and parental stress using the PSI score as an absolute value, as well as the results of the assessment for maternal attachment representations using the scores from the AMMI dimensions as absolute values. An additional analysis using mixed models will also be performed for exploratory purposes (linear models) to analyze the longitudinal aspects of the follow-up of the children included in the study. As described previously, a separate model will be performed for each Q-score.

All tests will be performed with a two-sided level of significance of 0.05.

## Discussion

The study will provide a better understanding of the attachment representations developed by extremely premature infants and the possible association between these attachment representations and maternal mental health, stress, and neurocognitive development. More knowledge and a better understanding could allow for individualized care at an early stage or even interventions from the neonatal period to improve the outcome of these vulnerable newborns.

The study also offers real advantages for the children and families included. An advisory consultation with the LIFT cohort’s usual partners (psychologist, child psychiatrist, etc.) will be offered if mental or interpersonal difficulties come to light during tests performed as part of the study.

The study is registered with ClinicalTrials.gov, identifier: NCT04304846 (retrospectively registered January 11, 2021). URL: https://clinicaltrials.gov/ct2/show/record/NCT04304846.

## Ethics and Dissemination

This study involving human participants qualifies as an interventional study with minimal risks and constraints in accordance with the decree of April 12, 2018 establishing the list of types of study mentioned in part 2 of Article L.1121-1 of the French Public Health Code, as an adapted care package may be offered following the results of the investigations carried out as part of the study.

The study file received a favorable opinion for the implementation of this research on February 18, 2020 - ID-RCB n°: 2019-A03352-55 (Dossier 2-20-007 id6699) 2°HPS. This study has received authorization from the French Data Protection Authority (CNIL) as per n° 920229.

The LIFT cohort received ethical approval from the Commission Nationale de l’Information et des Libertés (No. 851117) (37).

## Ethics Statement

The studies involving human participants were reviewed and approved by ID-RCB no. 2019-A03352-55 (File 2-20-007 id6699) 2°HPS. This study has received authorization from the French Data Protection Authority (CNIL) under no. 920229. Written informed consent to participate in this study was provided by the participants’ legal guardian/next of kin. Written informed consent was obtained from the individual(s), and minor(s)’ legal guardian/next of kin, for the publication of any potentially identifiable images or data included in this article.

## Author Contributions

ER, RS, FB, AV, RM, VR, JR, J-BM, and GG performed the design of the study. ER, VR, JR, J-BM, and GG contributed to the data analysis. All authors have read and approved the article, revised and wrote the manuscript.

## Conflict of Interest

The authors declare that the research was conducted in the absence of any commercial or financial relationships that could be construed as a potential conflict of interest.

## Publisher’s Note

All claims expressed in this article are solely those of the authors and do not necessarily represent those of their affiliated organizations, or those of the publisher, the editors and the reviewers. Any product that may be evaluated in this article, or claim that may be made by its manufacturer, is not guaranteed or endorsed by the publisher.
